# Pharmacopuncture for nerve entrapment syndrome: A protocol for systematic review

**DOI:** 10.1097/MD.0000000000031458

**Published:** 2022-11-25

**Authors:** Jin-Ho Jeong, Ji Hye Hwang

**Affiliations:** a Namsangcheon Korean Medicine Clinic, Seoul, Republic of Korea; b Department of Acupuncture & Moxibustion Medicine, College of Korean Medicine, Gachon University, Seongnam, Republic of Korea.

**Keywords:** acupoint injection, entrapment neuropathy, nerve entrapment, pharmacopuncture, protocol, systematic review

## Abstract

Nerve entrapment syndrome occurs when the nerves become compressed or entrapped and restricted. This study aims to evaluate the effectiveness and safety of pharmacopuncture in patients with nerve entrapment syndrome.

**Methods:**

A search will be conducted from inception to August 2022 using the following 11 electronic databases: MEDLINE, EMBASE, Cochrane Central Register of Controlled Trials, Allied and Complementary Medicine Database China National Knowledge Infrastructure, and 6 Korean databases. All randomized controlled trials (RCTs) evaluating pharmacopuncture treatment for various nerve entrapment syndromes will be considered, with no restrictions regarding the type of pharmacopuncture solution used. Two reviewers will perform the data extraction and quality assessment using a predefined data extraction form. The methodological quality of the included RCTs will be assessed using the Cochrane risk-of-bias tool.

**Results:**

This systematic review will provide high-quality evidence to determine the efficacy and safety of pharmacopuncture therapy for nerve entrapment syndrome.

**Conclusion:**

Our findings will be informative for patients with nerve entrapment syndrome, as well as clinicians, policymakers, and researchers.

## 1. Introduction

Nerve entrapment syndrome, also called entrapment neuropathy, is a group of peripheral nerve diseases that cause pain and abnormal sensations in the innervation area when a nerve is pathologically compressed in an anatomically narrow passage in its peripheral pathway.^[1–4]^ The most common entrapment neuropathy is carpal tunnel syndrome, with a lifetime risk of 10%, which increases to 84% in patients with diabetes.^[5,6]^ The second most common entrapment neuropathy is cubital tunnel syndrome.^[6,7]^ Peripheral nerve disorders considerably affect a patient’s functioning and quality of life.^[8]^

Generally, nonsurgical therapy for nerve entrapment syndrome is recommended for at least 3 months and comprises anti-inflammatory or analgesic drugs, splinting, avoiding aggravating activities or postures, physiotherapy, and topical steroid injections. Surgical intervention should be considered if symptoms worsen (despite nonsurgical treatment), or if severe symptoms or advanced findings (i.e., significant atrophy) are observed at the initial visit. Surgical management consists of nerve decompression, sometimes combined with other procedures, to provide a better pathway for nerves or to provide a bed for nerves (e.g., by transposition).^[9]^ Complementary and alternative medicine is gaining wide support as the demand for nonsurgical approaches increases. Pharmacopuncture is a complementary and alternative medicine treatment for nerve entrapment syndrome, mainly in Korean medicine (KM) and traditional Chinese medicine.

Pharmacopuncture (acupoint injection, herbal acupuncture, or aqua-acupuncture), a representative treatment of KM and traditional Chinese medicine, is a new type of acupuncture that combines conventional acupuncture and herbal extract injections based on meridians and pharmacology, and can provide the benefits of acupuncture and herbal medicine simultaneously.^[10–12]^ Currently, in traditional medicine, many herbal extracts are used for pharmacopuncture treatment of various diseases.^[13,14]^ Bee venom acupuncture is a pharmacopuncture that takes advantage of diluted bee venom instead of distilled herbal decoctions.^[15,16]^ Although pharmacopuncture has used for treating various diseases, it is most often applied in musculoskeletal disorders, and many studies on its therapeutic efficacy have been reported.^[13,14]^ The treatment of nerve entrapment syndrome with pharmacopuncture alone or combined with KM intervention has been reported, and clinical evidence suggests that pharmacopuncture treatment can reduce pain by relieving the entrapment of the peripheral nerves that dominate the area, thus improving the ischemic state of the nerve stem.^[17–20]^ However, data on the effectiveness of pharmacopuncture treatment for nerve entrapment syndrome are insufficient. In this study, we will review systematic randomized controlled trials (RCTs) to establish a therapeutic basis and expand clinical applications to evaluate the efficacy and safety of pharmacopuncture based on traditional medicine for nerve entrapment syndrome.

## 2. Methods

### 2.1. Study registration

The protocol for this systematic review was registered in the International Prospective Register of Systematic Reviews (registration number: CRD42022357344; https://www.crd.york.ac.uk/prospero/display_record.php?ID=CRD42022357344). The current protocol for this review complied with the preferred reporting items for systematic reviews and meta-analyses protocols.^[21]^

### 2.2. Data sources

The following 11 databases were searched from inception to August 2022: MEDLINE, EMBASE, the Cochrane Central Register of Controlled Trials, the Allied and Complementary Medicine Database, China National Knowledge Infrastructure, and 6 Korean databases (KoreaMed, the Korean Traditional Knowledge Portal, Oriental Medicine Advanced Searching Integrated System, the Research Information Service System, the Korean Studies Information Service System, and DBpia).

### 2.3. Types of studies

RCTs evaluating the effectiveness and safety of pharmacopuncture as a treatment for nerve entrapment syndrome will be included. All RCTs evaluating pharmacopuncture treatment for various nerve entrapment syndromes were considered. All types of pharmacopuncture (acupoint injection, aquapuncture, herbal aquapuncture) will be included. Non-randomized trials, animal or cell studies, case reports, literature research, and quasi-RCTs will be excluded, as will RCTs involving healthy participants. No language restrictions will be applied.

### 2.4. Types of participants

This study will include all participants with nerve entrapment syndrome. No restrictions will be placed in terms of sex, age, race, or nationality.

### 2.5. Types of interventions and controls

The treatment group received pharmacopuncture. There were no restrictions regarding the type of pharmacopuncture solution used. Bee venom is also included. Studies that assessed the combined effects of pharmacopuncture and other interventions will also be considered when an identical intervention was administered to both the pharmacopuncture and control groups. We will also exclude studies using pharmacopuncture solutions in combination with Western medicines and trials comparing pharmacopuncture with any type of control intervention, such as acupuncture, block therapy, or no treatment.

### 2.6. Types of outcome measures

#### 2.6.1. Primary outcomes.

The primary outcome will be pain severity measured by a visual analog scale, the Numeric Pain Rating Scale, or other validated pain scoring systems if visual analog scale was not used.

#### 2.6.2. Secondary outcomes.

The secondary outcomes will include improvement in neurological function, other scales or questionnaires evaluating pain or functional disability, treatment success rate, quality of life, recurrence rate, and complication rate.

### 2.7. Data extraction

**Figure 1.**
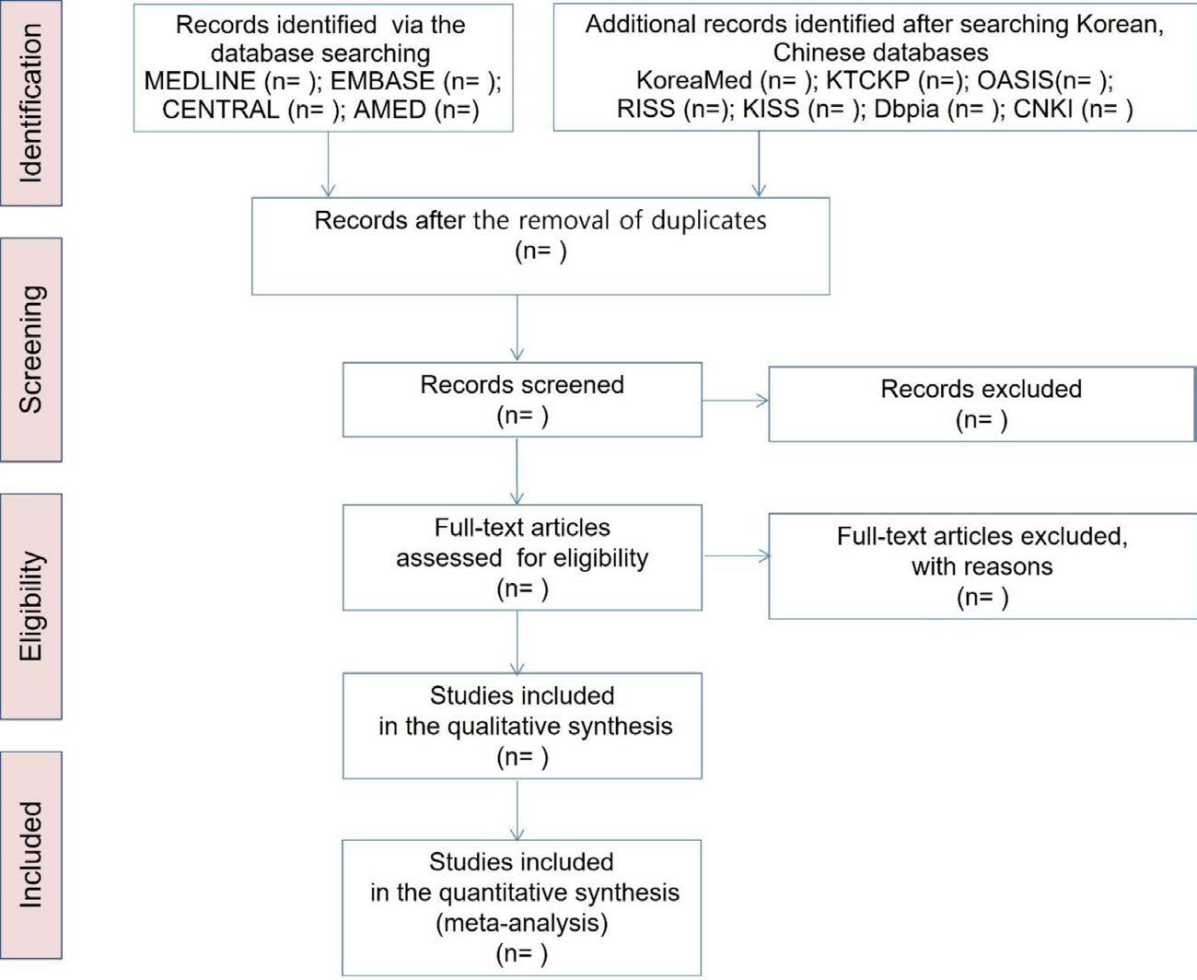
PRISMA flow diagram. AMED = allied and complementary medicine database, CENTRAL = Cochrane central register of controlled trials, CNKI = China national knowledge infrastructure, KISS = Korean studies information service system, KTCKP = Korean traditional knowledge portal, OASIS = oriental medicine advanced searching integrated system, PRISMA = preferred reporting items for systematic reviews and meta-analyses, RISS = research information service system.

We will review the eligibility of all searched articles for inclusion. In case of uncertainties, the authors will be contacted further information. Two reviewers (JHJ and JHH) will perform the data extraction and quality assessment using a predefined data extraction form. Data will be extracted, including author, age, country, year of publication, participant characteristics, interventions, randomization methods, blinding, control treatments, major outcomes, and adverse events. In research management, researchers use Endnote X9 (Fig. 1).

### 2.8. Quality assessment

The risk of bias will be assessed using the Cochrane Handbook risk-of-bias assessment tool version 5.1.0, in terms of random sequence generation, allocation concealment, blinding of participants and personnel, blinding of outcome assessment, incomplete outcome data, selective reporting, and other sources of bias. The assessment results will be presented using the scores “L,” “U,” and “H” for low, uncertain, and high risks of bias, respectively. If an article does not provide sufficient information for evaluation, we will contact the corresponding author for a clarification. Disagreements will be resolved via discussion among all authors.

### 2.9. Data synthesis and analysis

Differences between the intervention and control groups will be assessed. Mean differences (MDs) with 95% confidence intervals (CIs) will be used to measure the effects of treatment for continuous data. Other forms of data will be converted into MDs. For outcome variables on different scales, standard MDs with 95% CIs will be used. For dichotomous data, we will present treatment effects as relative risks with 95% CIs; other binary data will be converted into relative risk values.

All statistical analyses will be conducted using the Cochrane Collaboration’s software program Review Manager (version 5.3; The Nordic Cochrane Centre, The Cochrane Collaboration, Copenhagen, Denmark) for Windows. We will contact the corresponding authors of studies with missing information to acquire and verify the data whenever possible. When appropriate, we will pool the data across studies to conduct a meta-analysis using fixed or random effects. We will use the GRADEpro software from Cochrane Systematic Reviews to create a summary of findings table.

### 2.10. Ethics and dissemination

As the study will review published literature, it will involve no patient recruitment or personal data collection; thus, ethical approval is not required. The results of this systematic review will be published in a peer-reviewed journal and will be disseminated electronically and in print. This review will be updated to inform and guide healthcare practice.

## 3. Discussion

The main advantages of pharmacopuncture over traditional acupuncture are faster effects, easier dosage adjustments, and additional synergistic effect of acupuncture and herbal extracts. Pharmacopuncture for nerve entrapment syndrome is a nonsurgical treatment method associated with less pain and more comfortable access. Therefore, it is crucial to determine whether pharmacopuncture is a good option for patients.

Studies have shown that pharmacopuncture can be effective in reducing the symptoms of nerve entrapment syndrome; however, its efficacy has not been well-evaluated scientifically and systematically. Pharmacopuncture solutions for nerve entrapment and neurosensory disorders have also been developed in KM, but clinical evidence is lacking.^[22]^

In this systematic review, we aimed to evaluate the efficacy and safety of pharmacopuncture, including bee venom acupuncture, in patients with nerve entrapment syndrome and to provide further evidence. In KM, standardized pharmacopuncture treatment guidelines for each disease have not been fully established. Moreover, lack of data on clinical applications can impede health insurance inclusion and potentially lead to increased health-related costs.^[23]^

The evidence accumulated through our research will be useful to patients, practitioners, and health policymakers, enabling patients with nerve entrapment syndrome to receive appropriate pharmacopuncture treatment, and practitioners to determine the rationale for treatment decisions. Our findings may also contribute to establishing standardized guidelines for pharmacopuncture treatment, determining health insurance coverage for pharmacopuncture, and providing basic information for the standardization of pharmacopuncture treatment.

## Author contributions

JHH conceived the study and developed the criteria. JHH and JHJ searched the literature and analyzed the data. JHH wrote the protocol and JHH and JHJ revised the manuscript. All authors have read and approved the final manuscript.

**Data curation:** Ji Hye Hwang.

**Formal analysis:** Ji Hye Hwang.

**Funding acquisition:** Ji Hye Hwang.

**Investigation:** Jin-Ho Jeong.

**Methodology:** Ji Hye Hwang, Jin-Ho Jeong.

**Project administration:** Ji Hye Hwang.

**Supervision:** Ji Hye Hwang.

**Writing – original draft:** Ji Hye Hwang.
